# Evaluating the therapeutic efficacy of gastramide theranostics targeting cholecystokinin-2 receptors in a preclinical setting

**DOI:** 10.1039/d5ra08789a

**Published:** 2026-01-07

**Authors:** Marwa N. Rahimi, Jo-Anne Pinson, Joseph Hilton-Proctor, Jessica Van Zuylekom, Benjamin Blyth, Peter D. Roselt, Mohammad B. Haskali

**Affiliations:** a Department of Radiopharmaceutical Sciences, Cancer Imaging The Peter MacCallum Cancer Centre Victoria 3000 Australia mo.haskali@petermac.org; b Sir Peter MacCallum Department of Oncology, The University of Melbourne Victoria 3010 Australia; c Models of Cancer Translational Research Centre The Peter MacCallum Cancer Centre Victoria 3000 Australia

## Abstract

Cholecystokinin-2 receptors (CCK_2_R) are overexpressed in neuroendocrine tumors, making them attractive targets for radiopharmaceutical therapy. However, clinical CCK_2_R-targeting agents demonstrate limited therapeutic efficacy, with only a small fraction of patients achieving sufficient tumor uptake for effective treatment. This study presents the evaluation of novel gastramide theranostic compounds GA4 and GA13, designed with enhanced structural properties for superior CCK_2_R targeting. DOTA-GA4, DOTA-GA13, and the clinical standard CP04 were synthesized and radiolabeled with lutetium-177 (^177^Lu) for comprehensive evaluation. The gastramide analogues achieved superior radiochemical stability (≥99% *vs.* 94% for CP04) and demonstrated three-fold improved metabolic stability *in vivo*, with 87% intact peptides remaining compared to only 29% for CP04. [^177^Lu]Lu-DOTA-GA4 and [^177^Lu]Lu-DOTA-GA13 translated these stability advantages and increased binding affinity into exceptional therapeutic performance, reducing AR42J cell viability to 30% and 19% respectively compared to 50% for CP04. In xenograft studies, 20 MBq doses of gastramide compounds extended median survival by 22–23.5 days *versus* only 6 days for CP04. These results establish gastramide theranostics as an effective approach to CCK_2_R-targeted therapy, addressing current limitations and providing an improved treatment option for neuroendocrine tumors with substantially enhanced therapeutic outcomes.

## Abstract 2

Cholecystokinin-2 receptors (CCK_2_R) are overexpressed in neuroendocrine tumors, making them attractive targets for radionuclide therapy.

## Graphical abstract

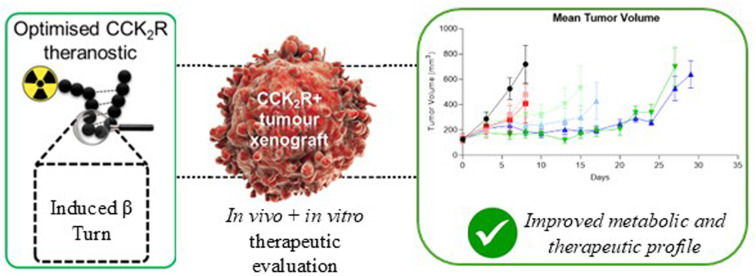

## Introduction

Peptide-based radiopharmaceuticals have become important therapeutics in oncology, delivering targeted radiation directly to cancer cells while ensuring rapid clearance from healthy tissue.^1,2^ Peptides that bind with high affinity to receptors overexpressed on tumor cells can be used for imaging when labeled with positron-emitting radionuclides such as gallium-68 (^68^Ga), or for therapy when labeled with cytotoxic radionuclides such as the beta emitter lutetium-177 (^177^Lu). This dual approach is termed theranostics and has shown promising clinical success.^3–7^

**Fig. 1:**
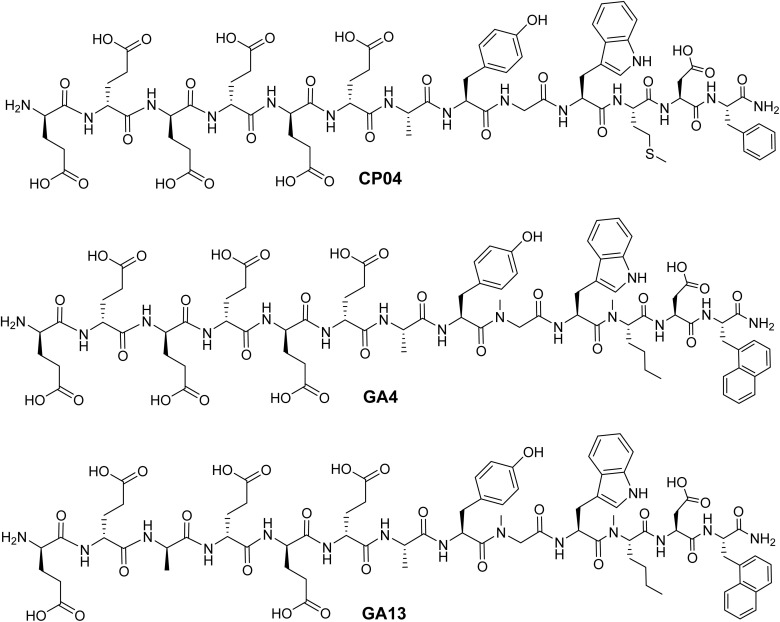
Chemical structures of CCK_2_R targeting peptides CP04, GA4, and GA13.

Cholecystokinin-2 receptors (CCK_2_R) are overexpressed in various cancers, including the majority of medullary thyroid carcinomas (MTC), more than half of small cell lung cancers (SCLC), and various neuroendocrine tumors (NETs).^8,9^ This expression pattern makes CCK_2_R an attractive target for nuclear medicine imaging and therapy. The endogenous CCK_2_R ligand gastrin and its 13-amino acid truncated form, minigastrin, have formed the basis for CCK_2_R theranostic development.^10,11^ CP04 (PP-F11) represents a well-established scaffold and is one of the most thoroughly studied ligands for CCK_2_R imaging in clinical settings (Fig. 1).^12,13^ The CP04 peptide was developed from the minigastrin structure by substituting the N-terminal l-glutamic acid residues with their d-isomers, resulting in enhanced metabolic stability and reduced renal uptake and retention.^14–16^

Our clinical experience with [^68^Ga]Ga-DOTA-CP04 PET/CT across 33 patients with various tumor types has demonstrated significant diagnostic utility while revealing opportunities for improvement. In medullary thyroid cancer, CP04-positive findings altered management in 33% of cases by enabling surgical resection, and CCK_2_R-targeted imaging has shown valuable outcomes in challenging atypical lung carcinoids.^13^ Notably, 89% of NETs patients showed CCK_2_R-positive disease despite many having negative somatostatin receptor 2 (SST-2R) expression.^13^

However, clinical findings have revealed important limitations of ^177^Lu-labeled CP04 for therapeutic applications. Many tumors demonstrated heterogeneous and relatively low uptake in CCK_2_R-positive tumors (average lesion SUV_max_ 6.8).^13^ Only two of eighteen patients with exceptionally high tumor uptake (SUV_max_ > 34) were considered suitable for [^177^Lu]Lu-DOTA-CP04 therapy, clearly indicating the need for improved agents that optimize tumor targeting while retaining minimal physiological uptake.^17,18^

MGS5 represents one promising approach to addressing these limitations. This minigastrin-derived truncated peptide has demonstrated clinical utility in both advanced medullary thyroid cancer and small cell lung cancer.^19,20^ In parallel, we have independently developed our innovative gastramide series of CCK_2_R-targeting compounds, which represents a distinct approach to this therapeutic space and will be comprehensively evaluated in this study.

Our approach led to the development of a novel library of CCK_2_R-binding peptides with enhanced structural properties designed to stabilize a biologically active β-hairpin structure.^21,22^ This methodology yielded GA4 and GA13, demonstrating superior potency both *in vitro* and *in vivo* when targeting CCK_2_R (Fig. 1).^17,21,22^ When coordinated with natural gallium, GA4 (IC_50_: 0.16 nM) and GA13 (IC_50_: 0.10 nM) achieved notable 14- and 24-fold improvements in binding affinity respectively compared to CP04 (IC_50_: 2.33 nM).^17^ The performance of GA4 has been validated through comprehensive metabolic stability studies, demonstrating excellent human serum stability over 24 hours with >97% intact peptide, and substantially extended half-lives of 25 h in mouse kidney homogenates (CP04 *t*½: 0.2 h) and 70 h in mouse liver homogenates (CP04 *t*½: 6 h).^1^

The present study assesses the efficacy of GA4 and GA13 peptides as therapeutic radiopharmaceuticals when conjugated to a DOTA chelator and labeled with the cytotoxic radionuclide ^177^Lu. As part of this evaluation, we examine the stability of these structures against radiolytic degradation, their *in vitro* toxicity, and their *in vivo* therapeutic efficacy, using the clinically established CP04 as a benchmark. This study is the first to systematically investigate gastramide peptides as β-hairpin structures for CCK_2_R-targeted radiotherapy.

## Methods

All chemicals were purchased from commercial suppliers at the highest available purity and used without further purification. Reagents, coupling agents, and amino acids were obtained from Sigma-Aldrich Australia, Merck (Germany), Combi-Blocks, ChemPep Inc, Chem-Impex International, Auspep Australia, and GL Biochem. Purified water was prepared using an in-house water purification system (Milli-Q® Advantage A10 system). Organic solvents (ACS grade) and anhydrous solvents included diethyl ether (Thermo Fisher Scientific), dichloromethane (DCM, Thermo Fisher Scientific), methanol (Thermo Fisher Scientific), acetonitrile (MeCN, Merck), and dimethylformamide (DMF, Thermo Fisher Scientific). DOTA was supplied by Combiblocks for DOTA coupling reactions.

YM022, a selective CCK_2_R antagonist,^23^ was used as a blocking agent in proliferation assays and purchased from Sigma-Aldrich (Cat. # SML0220). The Cell Counting Kit-8 (CCK-8) reagent for cell viability determination was obtained from Sigma Aldrich Australia (Cat. # 96992-100TESTS-F).

### Cell culture

AR42J rat pancreatic carcinoma cells were obtained from the American Type Culture Collection (ATCC, CRL-1492) and maintained in RPMI-1640 medium (Thermo Fisher Scientific, Cat. # 11875093) supplemented with 20% fetal bovine serum (FBS, Sigma-Aldrich Australia). Cells were cultured at 37 °C in a humidified atmosphere containing 5% CO_2_ and grown to approximately 90% confluence before use.

### Peptide synthesis and radiolabeling

The DOTA-modified peptides CP04, DOTA-GA4, and DOTA-GA13 were synthesized using standard protocols, and then purified, and isolated using established protocols to afford the target compounds in high chemical purity (>95% purity observed at 220 nm UV wavelength).^17,21^ Briefly, peptides were synthesized *via* Fmoc-based solid phase peptide synthesis (SPPS) using Rink amide resin on a CEM Liberty Blue microwave synthesizer at a 0.1 mmol scale. Fmoc groups were removed using a 20% piperidine/DMF solution containing oxyma under microwave irradiation. After rinsing with DMF, Fmoc-amino acids were coupled with DIC and oxyma in DMF, also using microwave irradiation. The peptide-resins were rinsed with dichloromethane prior to global deprotection and cleavage from the resin using a TFA-based cocktail for 2 hours.

Crude peptides were isolated and purified *via* preparative HPLC on a Phenomenex Gemini 5 µm C18 110 Å LC column (250 mm × 30 mm) using a gradient of MeCN: 0.1% (v/v) TFA. The gradient started at 30% MeCN for 1 minute, increased to 70% over 20 minutes, then to 90% for 2 minutes, and returned to 30%, all at a flow rate of 30 mL min^−1^. Quality control was conducted using analytical HPLC/MS on a Shimadzu HPLC system.

For the conjugation of DOTA to peptides, DOTA (1.5 eq. relative to the peptide) was pre-activated with NHS (2.25 eq.) using EDCI (2.25 eq.) and DIPEA (3 eq.) in anhydrous DMSO (approximately 500 µL) and sonicated at 50 °C for 30 minutes until dissolved. N-terminal free peptides GA4, GA13 and CP04 (1 eq.) were dissolved in a minimal volume of anhydrous DMSO (200–500 µL), shaken for 30 minutes at room temperature, and monitored by LC-MS. Upon completion, distilled water was added to create a 20 : 80 DMSO : water mixture, and the crude peptides were purified using reverse-phase HPLC employing the aforementioned conditions, yielding the final products as a white solids.

These DOTA-peptides were subsequently radiolabeled with ^177^Lu as detailed in the SI materials. As a negative control for *in vitro* assays, ^177^Lu-labeled diethylenetriaminepentaacetic acid (DTPA), an acyclic metal chelator with similar properties to DOTA but without peptide conjugation, was prepared. Comprehensive quality control testing was performed on all radiolabeled peptides, including assessment of final formulation appearance, pH, radionuclidic identity (half-life verification), radiochemical purity, and radiochemical identity (see Table S1 for complete specifications and results).

### *In vitro* proliferation assays

AR42J cells, a rat pancreatic tumor cell line with endogenous CCK_2_R expression, served as an established model for investigating CCK_2_R-targeting ligands.^21,22,24,25^ Cell-based assays were performed to assess cytotoxicity of ^177^Lu-labeled CP04, GA4, and GA13 and their subsequent impact on cell proliferation. [^177^Lu]Lu-DTPA served as a control to evaluate ^177^Lu effects that occur independently of CCK_2_R binding.

Cells were grown to log phase and divided into 1 × 10^6^ cell aliquots across three treatment groups for each ligand: “No Treatment”, “Treatment”, and “Treatment + Blocking”. After washing with cold binding buffer (RPMI-1640 + 1% FCS), the “Treatment + Blocking” groups were pre-treated with YM022 (500 µL, 1 µM) for 1 hour at room temperature with gentle rotation, while “Treatment” groups received binding buffer only (500 µL) under identical conditions. The radiolabeled ligands [^177^Lu]Lu-CP04, [^177^Lu]Lu-DOTA-GA4, [^177^Lu]Lu-DOTA-GA13, and control [^177^Lu]Lu-DTPA were prepared at 5 MBq per mL (100–115 nM) and added to appropriate aliquots (500 µL each). Following 4 hours of gentle rotation at room temperature, cells were washed with ice-cold binding buffer to remove unbound ligand, resuspended in growth media, and plated at 750 cells per well in flat-bottomed 96-well plates (100 µL). Cell proliferation was monitored daily over 10 days using the CCK-8 colorimetric assay. The reagent was diluted 1 : 10 in binding buffer, added to 96-well plates, and incubated at 37 °C for 1 hour before reading absorbance at 450 nm.

### *In vivo* metabolism studies

All animal experiments were conducted in accordance with the Australian Code for the Care and Use of Animals for Scientific Purposes (8th Edition, 2013) and approved by the Peter MacCallum Cancer Centre Animal Experimentation Ethics Committee (Ethics application number AEEC E636). Naïve female BALB/c nude mice were injected with 15 MBq of ^177^Lu-labeled CP04, GA4, and GA13 (50 µL injections). After 15 minutes, blood (500 µL) and urine (20 µL) was collected.

Proteins in blood were precipitated out in equal volume of acetonitrile (500 µL) which was vortexed. Samples were centrifuged at 14 000 rpm for 3.5 minutes. Supernatant (50 µL) was added to MilliQ water (50 µL) and transferred to LC-MS glass vials with inserts and analyzed using quantitative radio-HPLC.

The collected urine was diluted 1 : 5 in ultrafiltered MilliQ water. Diluted urine (100 µL) was added to acetonitrile (100 µL) to precipitate proteins. Samples were centrifuged at 14 000 rpm for 3.5 minutes. Supernatant (100 µL) was transferred to LC-MS glass vials with inserts and analyzed using quantitative radio-HPLC.

### *In vivo* therapy study

Female BALB/c nude mice (6–9 weeks old, 14–20 g) were sourced from Animal BioResources (Moss Vale, New South Wales) and subcutaneously implanted with 3 × 10^6^ AR42J cells with high endogenous CCK_2_R expression. Cells were injected in a 1 : 1 Matrigel (Corning) : PBS mixture (100 µL) on the right flank.

Following inoculation, mice were monitored with thrice-weekly measurements of body weight and tumor dimensions using electronic calipers. Tumor volume (mm^3^) was calculated using the formula: length × width × height × π/6. Once tumors reached appropriate volumes, animals were randomized into groups of six and administered intravenous injections of either vehicle control, [^177^Lu]Lu-CP04, [^177^Lu]Lu-DOTA-GA4, or [^177^Lu]Lu-DOTA-GA13 at doses of 10 MBq or 20 MBq.

Mouse weights (Tables S5 and S6) and tumor volumes (Tables S7 and S8) are detailed in SI materials. Mice were euthanized when tumors reached the endpoint volume (>1200 mm^3^) or met other predefined humane criteria as outlined in the Australian Code of Practice for the Care and Use of Animals for Scientific Purposes.

### Statistics

*In vitro* proliferation assays were conducted using pairwise comparisons between treatment groups with multiple two-tailed *t*-tests and Holm–Šídák correction. For *in vivo* survival analyses, Kaplan–Meier curves were compared using the log-rank (Mantel–Cox) test. Hazard ratios and confidence intervals were not calculated, as the focus was on assessing relative therapeutic efficacy and survival benefits. Data were analyzed using GraphPad Prism Software version 10.0.3 (GraphPad Software, California, USA).

## Results and discussion

### Gastramide peptides [^177^Lu]Lu-DOTA-GA4 and [^177^Lu]Lu-DOTA-GA13 demonstrate enhanced radiostability compared to [^177^Lu]Lu-CP04

All DOTA-modified peptides were synthesized using previously established methods, yielding the target compounds with >95% purity.^17,21,22^ Detailed methods for synthesis, DOTA coupling and subsequent radiolabeling with ^177^Lu can be found in SI materials. For radiolabeling, non-carrier-added [^177^Lu]LuCl_3_ was obtained from the Australian Nuclear Science and Technology Organisation (ANSTO). Radiolabeling with ^177^Lu was performed using a buffer composed of 0.4 M ammonium acetate, 0.24 M gentisic acid, 0.067 M l-methionine, and 0.05 M sodium ascorbate, dissolved in a 4 : 1 mixture of H_2_O and EtOH, achieving over 98% radiochemical incorporation and radiochemical purity of ≥94% (Table S1). The inclusion of gentisic acid, l-methionine, and sodium ascorbate ensured that the final formulation remained stable against radiolytic breakdown for at least 48 hours post-synthesis, with purities ≥90%. l-Methionine was particularly crucial for stabilizing [^177^Lu]Lu-CP04, preventing the methionine in the sequence from undergoing radiolytic oxidation to the corresponding sulfoxide.

Comprehensive quality control assessment was conducted on each radiolabeled peptide, evaluating final formulation appearance, pH, and radiochemical purity (Table S1). All radioligands consistently met established quality control criteria throughout the study, including radiochemical purity verification by both HPLC and TLC analysis.

The methionine residue in CP04 (Fig. 1) is susceptible to radiolytic oxidation at elevated temperatures during radiolabeling, compromising radiochemical purity as determined by HPLC analysis.^26^ This represents a well-documented challenge, with the sulfoxide oxidation product typically comprising 5–10% of the final radioligand.^26–29^ Our radiosynthesis of [^177^Lu]Lu-CP04 yielded comparable results, achieving 94% radiochemical purity despite the use of stabilizing agents (Table S1). The oxidized product accounted for approximately 6% of the final material (Fig. S1, and Table S2).

The reactive methionine in CP04 was substituted with the non-oxidizing isosteric analogue norleucine, which was *N*-methylated at the amide bond to induce a turn motif in the peptide sequence during the development of GA4 and GA13.^17,21^ When conjugated to DOTA and radiolabeled with either ^68^Ga or ^177^Lu, these gastramide analogues consistently demonstrated improved radiochemical purity (≥99%) compared to CP04 (Table S1, Fig. S2 and S3).^17,21^ While all radioligands in this study met established preclinical quality standards, GA4 and GA13 offer notable advantages for clinical translation through their enhanced radiochemical stability and reduced degradation, making them promising candidates for clinical development.

### [^177^Lu]Lu-CP04 is more readily metabolized *in vivo* compared to gastramide radioligands GA4 and GA13

Radiolabeled peptides were evaluated for metabolic stability by quantifying the amount of intact radiopeptide present in naïve BALB/c mouse blood and urine 15 minutes after injection. The percentage of intact peptide was determined by radio-HPLC analysis, quantifying the relative area under the curve (AUC) of peaks corresponding to the intact and metabolized peptide. This provides an important assessment of peptide stability after traversing murine *in vivo* excretory pathways.

The analysis of blood samples showed that no detectable signals were found for [^177^Lu]Lu-CP04, [^177^Lu]Lu-DOTA-GA4, or [^177^Lu]Lu-DOTA-GA13 (see Fig. S5–S7). This suggests that all three peptides are cleared from the bloodstream rapidly, with no radiometabolites detected after 15 minutes.

[^177^Lu]Lu-CP04 was readily metabolized *in vivo* at 15 minutes post-injection, the majority of which was detected as a single unresolved peak at the solvent front (Fig. S8). Along with small peaks detected at 6.27 and 8.23 minutes (Table 1), the metabolized peptide accounted for 71.0 ± 10.8% of the peptide detected by radio-HPLC. GA4 and GA13 were developed to address the limitations identified in earlier investigations.^17^ The structural modifications introduced in GA4 and GA13 resulted in substantial improvement in metabolic stability *in vivo*: [^177^Lu]Lu-DOTA-GA4 and [^177^Lu]Lu-DOTA-GA13 were found 87.0 ± 2.2% and 86.7 ± 2.4% intact respectively in mouse urine (Table 1). This represents an approximate three-fold improvement in stability of gastramide peptides compared to CP04.

**Table 1:** *In vivo* peptide metabolism as determined by peptide % intact in urine at 15 minutes post-injection. Molar activity, retention, % composition of ^177^Lu-labeled CP04 and gastramide peptides GA4, and GA13 (*n* = 3)

	Molar activity^a^ (*A*_M_)		Intact peptide	Metabolized peptide
[^177^Lu]Lu-CP04	27.68 MBq per nmol	Retention time (min)^b^	5.72	1.59, 6.27, 8.23
		Composition (%)	29.0 ± 10.8	71.0 ± 10.8
[^177^Lu]Lu-DOTA-GA4	21.19 MBq per nmol	Retention time (min)^c^	12.47	1.57
		Composition (%)	87.0 ± 2.2	13.0 ± 2.2
[^177^Lu]Lu-DOTA-GA13	19.93 MBq per nmol	Retention time (min)^c^	12.46	1.58
		Composition (%)	86.7 ± 2.4	13.3 ± 2.4

a Molar activity at end of synthesis.

b HPLC conditions: Kinetex XB C18 column (5 µm, 100 Å, 250 × 4.60 mm) eluted at 1 mL min^−1^ with a gradient of MeCN: 0.05% (v/v) TFA, starting at 25% MeCN for 1 min, increased to 35% over 9 minutes, then instantly increased to 90% and maintained for 2 minutes before returning to 25% MeCN.

c HPLC conditions: Kinetex XB C18 column (5 µm, 100 Å, 250 × 4.60 mm) eluted at 1 mL min^−1^ with a gradient of MeCN: 0.05% (v/v) TFA, starting at 5% MeCN for 2 min, increased to 60% over 13 minutes, then instantly increased to 90% and maintained for 2.5 minutes before returning to 5% MeCN.

In summary, the analysis indicated that the radiometabolites of the three ligands appeared as unresolved peaks at the solvent front, representing rapidly generated polar, low-molecular-weight peptide fragments, with no intermediate or late-eluting radioactive species observed. The observed differences in the proportion of intact peptide between CP04 and the GA analogues underscore the enhanced resistance of the latter to proteolytic degradation.

### ^177^Lu-labeled GA4 and GA13 demonstrated enhanced cytotoxicity in AR42J cells compared to [^177^Lu]Lu-CP04

This proliferation assay was designed to assess the relative cytotoxicity and proliferation effects of CP04 and the gastramide peptides GA4 and GA13 in AR42J cells, a rat cell line that endogenously expresses CCK_2_R. This cell line is widely employed for preclinical evaluation of minigastrin-based imaging and therapeutic constructs and allows for direct comparison against historical CP04 and other CCK_2_R targeting ligands.

**Fig. 2:**
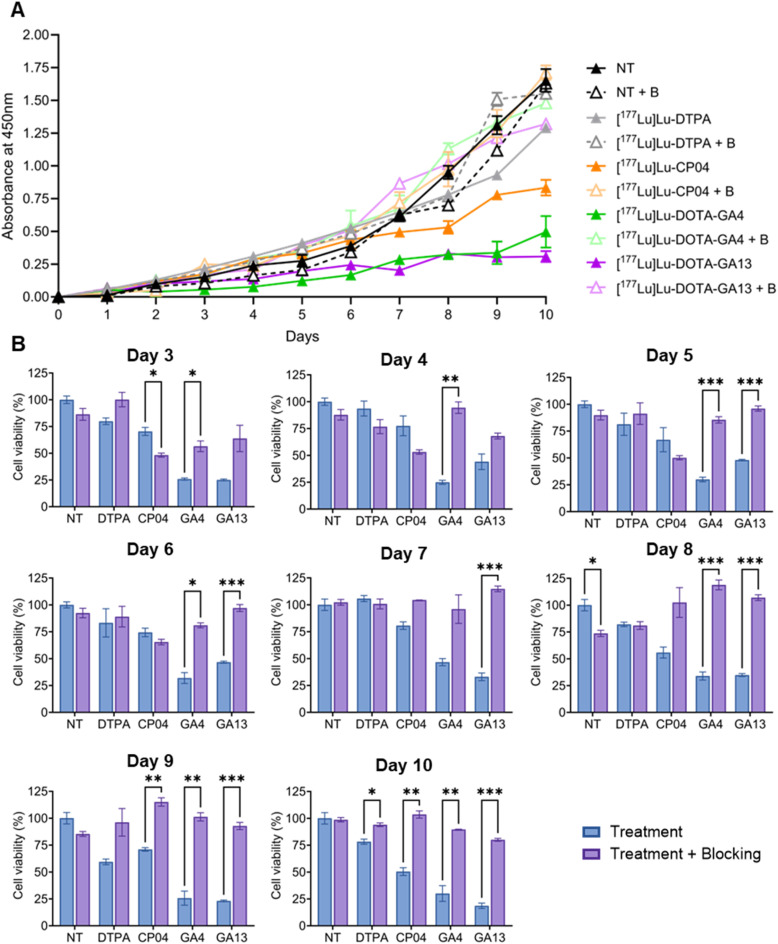
(A) AR42J growth rates for no treatment (NT) ± blocking (B), and treated with [^177^Lu]Lu-DTPA, [^177^Lu]Lu-CP04, [^177^Lu]Lu-DOTA-GA4, and [^177^Lu]Lu-DOTA-GA13 over 10 days. Each ligand was evaluated in “No Treatment”, “Treatment”, and “Treatment + Blocking” cohorts. In blocking experiments, cells were pre-blocked with YM022. All treatments in triplicate, *n* = 3. Error bars represent standard error of the mean (SEM). (B) Pairwise comparison of treatments analyzed on days 3–10. Cell viability determined by normalizing to “No Treatment” control cohort. *P*-Value threshold determined using the Holm-Šídák method and stratified according to APA style: *p* < 0.033 (*), *p* < 0.002 (**), *p* < 0.001 (***). Only *p* values greater than 0.05 have been indicated.

By treating AR42J cells with CCK_2_R-targeting radioligands and monitoring cell viability over 10 days, the relative cytotoxicity of these agents was quantitatively determined. [^177^Lu]Lu-DTPA served as a negative control to assess toxicity from non-specific ^177^Lu exposure without CCK_2_R targeting. To confirm receptor-specific binding, YM022 blocking treatment groups were included for all radioligands, with raw absorbance values plotted alongside untreated controls. AR42J cells exhibit slow proliferation kinetics with a doubling time of approximately three days, as demonstrated in the untreated control groups across all treatments (Fig. 2). Pairwise comparison of “Treatment” and “Treatment + Blocking” was performed to evaluate differences between these treatments, starting from day 3 as cells were stabilizing on days 1–2 (Fig. 2). After initial culture stabilization, cells exhibited the expected exponential growth from day 4 onward, reaching maximum density by day 10. This timepoint was selected as the assay endpoint to avoid exceeding the upper detection limit of the CCK-8 colorimetric assay.

The effect of YM022 treatment on cell proliferation was observed in growth rate graphs and quantified in pairwise comparison of treatments (Fig. 2). Compared to “No treatment” cohorts, YM022-treated cells in “No Treatment + Blocking” displayed decreased cell proliferation on days 3–6 and 8–9, with a significant difference observed on day 8 (73.6 ± 4.1% cell viability *versus* 100 ± 7.5% “No Treatment”, *p* = 0.012). The anti-proliferative effects of YM022 have been previously investigated, with studies showing YM022 inhibited both gastrin-induced and basal proliferation in various cell types.^30^ AR42J cells are derived from rat pancreatic tumors and naturally express CCK_2_R, which respond to gastrin and cholecystokinin (CCK) by activating pathways associated with cell growth and survival.^31^ Therefore, blocking CCK_2_R with YM022 results in reduced cell proliferation.

[^177^Lu]Lu-DTPA treatment, with or without YM022 blocking, showed no significant impact on cell growth compared to controls throughout the assay, demonstrating that CCK_2_R-specific targeting is essential for ^177^Lu therapeutic efficacy (Fig. 2).

[^177^Lu]Lu-CP04 exhibited a growth profile similar to untreated and [^177^Lu]Lu-DTPA controls during the initial phase (days 0–5), with modest growth inhibition beginning to emerge by day 7. In contrast, the gastramide radioligands ([^177^Lu]Lu-DOTA-GA4 and [^177^Lu]Lu-DOTA-GA13) demonstrated rapid and pronounced growth inhibition evident as early as day 3.

Cell viability following [^177^Lu]Lu-DTPA treatment remained statistically equivalent to untreated controls on each recorded day (Fig. 2). Over ten days, cell viability of [^177^Lu]Lu-DTPA-treated cells was 109.0 ± 33.1% and 93.0 ± 11.5% for “Treatment” and “Treatment + Blocking” respectively.

Quantitative cell viability analysis revealed distinct therapeutic profiles among the radioligands tested. AR42J cells treated with [^177^Lu]Lu-CP04 had cell viability greater than 75% on days 1–8, indicating limited observable effect on cell proliferation. The cytotoxic activity of CP04 became pronounced on days 9 and 10, with cell viability of 71.0 ± 2.4% and 50.5 ± 5.1% respectively compared to “No Treatment” controls.

In contrast, [^177^Lu]Lu-DOTA-GA4's cytotoxic effect was observed earlier, with 25.8 ± 1.4% and 24.9 ± 2.6% cell viability on days 3 and 4 respectively compared to untreated controls. [^177^Lu]Lu-DOTA-GA4 continued to significantly impact cell proliferation on subsequent days, with an average cell viability of 33.7% across days 5–9. Specificity of [^177^Lu]Lu-DOTA-GA4-dependent cytotoxicity was confirmed by statistically significant differences between GA4 “Treatment” and “Treatment + Blocking” cohorts (Fig. 2). [^177^Lu]Lu-DOTA-GA4 consistently impacted AR42J cell proliferation throughout the study, ultimately reaching cell viability of 30.0 ± 10.4% on day 10. At study conclusion, [^177^Lu]Lu-DOTA-GA4 treatment decreased cell viability 3.3-fold compared to the “No Treatment” group, and 1.6-fold more effectively than [^177^Lu]Lu-CP04 treatment.

[^177^Lu]Lu-DOTA-GA13 also displayed strong anti-proliferative effects on cell growth, demonstrating 25.0 ± 1.2% cell viability on day 3, with a marginal increase on day 4 at 44.1 ± 10.3%. On day 10, [^177^Lu]Lu-DOTA-GA13 was the most cytotoxic agent for AR42J cells, with cell viability of 18.6 ± 3.6%. This represents a 2.8-fold greater cytotoxic effect than [^177^Lu]Lu-CP04.

Both GA4 and GA13 candidates exhibited significant cytotoxicity, impacting AR42J cell proliferation. Additionally, toxicity was confirmed to be CCK_2_R-specific, with cell viability on day 10 at 30.0 ± 10.3% and 18.6 ± 3.6% for GA4 and GA13 “Treatment” respectively compared to 89.6 ± 0.4% and 80.0 ± 1.9% “Treatment + Blocking” for the gastramide peptides (Fig. 2).

### ^177^Lu-labeled gastramide radioligands demonstrate enhanced tumor control and survival benefits in the AR42J xenograft model

**Fig. 3:**
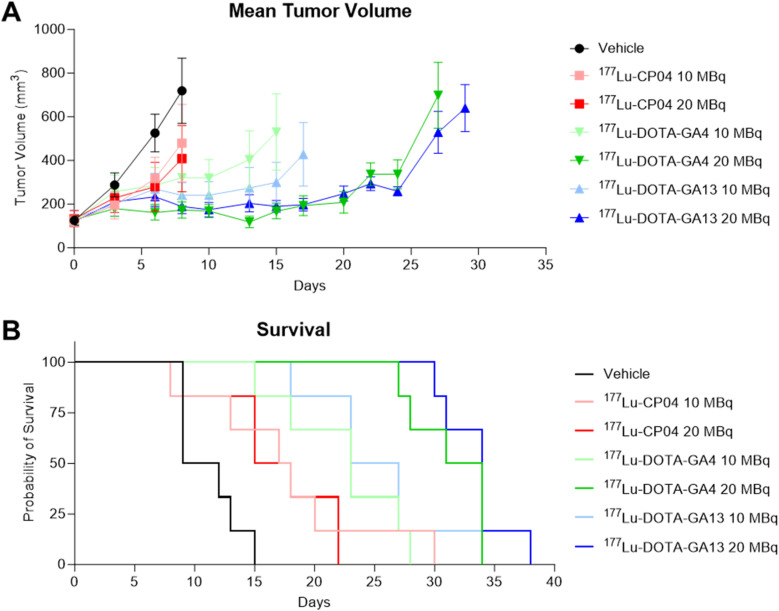
(A) Mean tumor (AR42J) volumes of mice treated with either 10 MBq or 20 MBq of [^177^Lu]Lu-CP04, [^177^Lu]Lu-DOTA-GA4, and [^177^Lu]Lu-DOTA-GA13 compared to vehicle (*n* = 6 per group). (B) Kaplan–Meier survival plots showing probability of survival in the 10 MBq and 20 MBq treatment groups compared to vehicle.

Cohorts of six AR42J xenograft mice received a single intravenous injection of either saline vehicle or ^177^Lu-labeled peptides (CP04, GA4, and GA13) at doses of 10 MBq or 20 MBq on day 0, with tumor growth monitored over 40 days (Fig. 3). Mice receiving vehicle control demonstrated rapid tumor progression, with mice reaching endpoint from day 7. [^177^Lu]Lu-CP04 treatment produced modest tumor growth suppression at both 10 MBq and 20 MBq doses. Comparison of therapeutic effects at 10 MBq and 20 MBq showed no significant difference in median survival (*p* = 0.96).

[^177^Lu]Lu-DOTA-GA4 treatment resulted in an average tumor volume of 405 mm^3^ at day 13 for the 10 MBq cohort, while the 20 MBq treatment resulted in average 119 mm^3^ tumor volume, representing a 3.4-fold reduction. Treatment with [^177^Lu]Lu-DOTA-GA13 yielded average tumor volumes of 274 mm^3^ at day 13 for the 10 MBq dose and 204 mm^3^ for the 20 MBq treatment. These results demonstrate that gastramide analogues achieved superior therapeutic efficacy compared to [^177^Lu]Lu-CP04 (Fig. 3).

Mice treated with 10 MBq of the gastramide peptides began reaching endpoint from day 15 onwards. In contrast, 20 MBq-treated mice displayed substantial tumor suppression even at day 20, with average tumor sizes of 209 mm^3^ and 248 mm^3^ for [^177^Lu]Lu-DOTA-GA4 and [^177^Lu]Lu-DOTA-GA13 respectively. These results demonstrate dose-dependent improvements in tumor control for gastramide radioligands. The enhanced tumor control translated into significant survival benefits. In the 10 MBq treatment group, median survival was prolonged by 12.5 days ([^177^Lu]Lu-DOTA-GA4) and 14.5 days ([^177^Lu]Lu-DOTA-GA13) compared to vehicle-treated mice (Table 2). In the 20 MBq treatment group, median survival was prolonged by 22 days ([^177^Lu]Lu-DOTA-GA4) and 23.5 days ([^177^Lu]Lu-DOTA-GA13) compared to vehicle-treated mice (Fig. 3, and Table 2). Furthermore, the difference between survival of mice treated with [^177^Lu]Lu-DOTA-GA4 and [^177^Lu]Lu-DOTA-GA13 at 10 MBq (*p* = 0.42) and 20 MBq (*p* = 0.32) was not statistically significant, indicating both agents are similarly effective at CCK_2_R-specific tumor suppression (Table 2). While the present data demonstrates substantial preclinical efficacy, these findings require confirmation in human-CCK_2_R models prior to clinical translation.

**Table 2:** Median survival (days) of mice bearing AR42J tumors for 10 MBq or 20 MBq of [^177^Lu]Lu-CP04, [^177^Lu]Lu-DOTA-GA4 or [^177^Lu]Lu-DOTA-GA13 compared to vehicle

	10 MBq treatment	20 MBq treatment	a *P*-Values
Vehicle	10.5 days		
[^177^Lu]Lu-CP04	17.5 days	16.5 days	0.96 (ns)
[^177^Lu]Lu-DOTA-GA4	23.0 days	32.5 days	0.005 (**)
[^177^Lu]Lu-DOTA-GA13	25.0 days	34.0 days	0.022 (*)
b *P*-values	0.42 (ns)	0.32 (ns)	

a *P*-Values determined by comparing same ligand at 10 MBq and 20 MBq treatments using log-rank (Mantel–Cox) test.

b *P*-Values determined by comparing [^177^Lu]Lu-DOTA-GA4 and [^177^Lu]Lu-DOTA-GA13 at different doses using log-rank (Mantel–Cox) test.

These results demonstrate dose-dependent improvements in both tumor control and survival for the gastramide radioligands, with [^177^Lu]Lu-CP04 showing moderate therapeutic response compared to the superior efficacy and survival benefits of the gastramide analogues. No treatment-related weight loss or early morbidity were observed in any cohort, including at the highest administered activity (Tables S5 and S6). Given that GA4 and GA13 retain the same radionuclide (^177^Lu) and chelator (DOTA) as CP04, their toxicity profile is expected to be comparable to established minigastrin-based radiotherapeutics evaluated at similar doses. Formal organ-specific and chronic toxicity assessments will be undertaken in future therapy-enabling studies as these candidates progress toward clinical translation.

## Conclusion

This preclinical evaluation demonstrates that gastramide radioligands [^177^Lu]Lu-DOTA-GA4 and [^177^Lu]Lu-DOTA-GA13 exhibit improved performance compared to the clinically established [^177^Lu]Lu-CP04 for CCK_2_R-targeted radiotherapy. In contrast to CP04, which is susceptible to radiolytic oxidation during ^177^Lu labeling and rapid enzymatic degradation *in vivo*, the gastramide analogues exhibited improved radiochemical purity, resistance to radiolytic breakdown (≥99% *vs.* 94% purity), and a three-fold improvement in metabolic stability *in vivo*. The gastramide analogues achieved improved cytotoxic potency (18.6–30.0% *vs.* 50.5% cell viability), and superior therapeutic efficacy with median survival extensions of 22–23.5 days for the gastramide compounds compared to 6 days for CP04 following treatment with 20 MBq of radioligand.

These findings validate our β-hairpin stabilization strategy and highlight GA4 and GA13 as promising candidates for the treatment of CCK_2_R-positive malignancies. The structural features designed for conformational control not only enhance stability and tumor uptake but also represent significant advancements in peptide design. The synthetic accessibility, favorable stability profile, and therapeutic performance of GA4 and GA13 position these agents comparably to clinically translated CCK_2_R ligands like CP04 and MGS5, paving the way for future therapeutic applications.

## Conflicts of interest

The authors declare no competing financial interests other than a provisional patent application covering the gastramide compounds and methods described in this work. All other aspects of this research were conducted without any commercial or financial relationships that could be construed as potential conflicts of interest.

## Abbreviations

CCK_2_R: cholecystokinin 2 receptor
DOTA: 1,4,7,10-tetraazacyclododecane-1,4,7,10-tetraacetic acid
PBS: phosphate buffered saline
HEPES: 4-(2-hydroxyethyl)-1-piperazineethanesulfonic acid
HPLC: high-performance liquid chromatography
YM022: (*R*)-*N*-[2,3-Dihydro-1-[2-(2-methylphenyl)-2-oxoethyl]-2-oxo-5-phenyl-1*H*-1,4-benzodiazepin-3-yl]-*N*′-(3-methylphenyl)-urea
DMEM: Dulbecco's modified eagle medium
TFA: trifluoroacetic acid
TIPS: triisopropylsilane
DIPEA: *N*,*N*-diisopropylethylamine
NHS: *N*-hydroxysuccinimide
EDCI: *N*-(3-dimethylaminopropyl)-*N′*-ethylcarbodiimide hydrochloride
DIC: *N*,*N*-diisopropylcarbodiimide
DCM: dichloromethane
MeCN: acetonitrile
DMSO: dimethylsulfoxide
NaOH: sodium hydroxide

## Supplementary Material

RA-016-D5RA08789A-s001

## Data Availability

The data supporting this article have been included as part of the supplementary information (SI). Supplementary information: SI figures and tables. See DOI: https://doi.org/10.1039/d5ra08789a.

## References

[cit1] Nhàn N. T. T., Yamada T., Yamada K. H. (2023). Peptide-Based Agents for Cancer Treatment: Current Applications and Future Directions. Int. J. Mol. Sci..

[cit2] Sgouros G., Bodei L., McDevitt M. R., Nedrow J. R. (2020). Radiopharmaceutical therapy in cancer: clinical advances and challenges. Nat. Rev. Drug Discov..

[cit3] Zhang H., Koumna S., Pouliot F., Beauregard J.-M., Kolinsky M. (2021). PSMA theranostics: current landscape and future outlook. Cancers.

[cit4] Rahbar K., Afshar-Oromieh A., Jadvar H., Ahmadzadehfar H. (2018). PSMA theranostics: current status and future directions. Mol. Imaging.

[cit5] Jeitner T. M., Babich J. W., Kelly J. M. (2022). Advances in PSMA theranostics. Transl. Oncol..

[cit6] Mallak N., O'Brien S. R., Pryma D. A., Mittra E. (2024). Theranostics in neuroendocrine tumors. Cancer J..

[cit7] Ichikawa Y., Kobayashi N., Takano S., Kato I., Endo K., Inoue T. (2022). Neuroendocrine tumor theranostics. Cancer Sci..

[cit8] Klingler M., Decristoforo C., Rangger C., Summer D., Foster J., Sosabowski J. K., von Guggenberg E. (2018). Site-specific stabilization of minigastrin analogs against enzymatic degradation for enhanced cholecystokinin-2 receptor targeting. Theranostics.

[cit9] Novak D., Tomašič T., Krošelj M., Javornik U., Plavec J., Anderluh M., Kolenc Peitl P. (2021). Radiolabelled CCK2R antagonists containing PEG linkers: design, synthesis and evaluation. ChemMedChem.

[cit10] Brom M., Joosten L., Oyen W., Boerman O., Gotthardt M. (2009). Development of a Ga-68-labelled gastrin-based PET tracer for the detection of CCK2/gastrin receptor positive neuroendocrine tumors. J. Nucl. Med..

[cit11] Béhé M., Behr T. M. (2002). Cholecystokinin-B (CCK-B)/gastrin receptor targeting peptides for staging and therapy of medullary thyroid cancer and other CCK-B receptor expressing malignancies. J. Pept. Sci..

[cit12] Lezaic L., Erba P. A., Decristoforo C., Zaletel K., Mikolajczak R., Maecke H., Maina T., Konijnenberg M., Kolenc P., Trofimiuk-Müldner M., Przybylik-Mazurek E., Virgolini I., de Jong M., Fröberg A. C., Rangger C., Di Santo G., Skorkiewicz K., Garnuszek P., Solnica B., Nock B. A., Fedak D., Gaweda P., Hubalewska-Dydejczyk A. (2023). [(111)In]In-CP04 as a novel cholecystokinin-2 receptor ligand with theranostic potential in patients with progressive or metastatic medullary thyroid cancer: final results of a GRAN-T-MTC Phase I clinical trial. EJNMMI Res..

[cit13] Kong G., Sutherland D., Roselt P., Hicks R., Haskali M., Akhurst T. (2023). Evaluation of [68Ga] Ga-DOTA-CP04 imaging in Cholecystokinin-2 receptors (CCK-2R) positive tumors. J. Nucl. Med..

[cit14] Ocak M., Helbok A., Rangger C., Peitl P. K., Nock B. A., Morelli G., Eek A., Sosabowski J. K., Breeman W. A., Reubi J. C. (2011). Comparison of biological stability and metabolism of CCK2 receptor targeting peptides, a collaborative project under COST BM0607. EJNMMI Res..

[cit15] Aloj L., Aurilio M., Rinaldi V., D'ambrosio L., Tesauro D., Peitl P. K., Maina T., Mansi R., von Guggenberg E., Joosten L. (2011). Comparison of the binding and internalization properties of 12 DOTA-coupled and 111 In-labelled CCK2/gastrin receptor binding peptides: a collaborative project under COST Action BM0607. EJNMMI Res..

[cit16] Peitl P. K., Tamma M., Kroselj M., Braun F., Waser B., Reubi J. C., Dolenc M. S., Maecke H. R., Mansi R. (2015). Stereochemistry of amino acid spacers determines the pharmacokinetics of 111In-DOTA-Minigastrin analogues for targeting the CCK2/gastrin receptor. Bioconjugate Chem..

[cit17] Rahimi M. N., Corlett A., Van Zuylekom J., Sani M. A., Blyth B., Thompson P., Roselt P. D., Haskali M. B. (2024). Precision peptide theranostics: developing N-to C-terminus optimized theranostics targeting cholecystokinin-2 receptor. Theranostics.

[cit18] Behr T. M., Jenner N., Béhé M., Angerstein C. (1999). Radiolabeled peptides for targeting cholecystokinin-B/gastrin receptor-expressing tumors. J. Nucl. Med..

[cit19] Di Santo G., Santo G., Martinovic V., Wolf D., Pircher A., Sviridenko A., Löffler-Ragg J., von Guggenberg E., Virgolini I. (2024). Cholecystokinin-2 receptor targeting by [(68)Ga]Ga-DOTA-MGS5 PET/CT in a patient with extensive disease small cell lung cancer. EJNMMI Res..

[cit20] von Guggenberg E., Uprimny C., Klinger M., Warwitz B., Sviridenko A., Bayerschmidt S., di Santo G., Virgolini I. J. (2023). Preliminary Clinical Experience with Cholecystokinin-2 Receptor PET/CT Using the (68)Ga-Labeled Minigastrin Analog DOTA-MGS5 in Patients with Medullary Thyroid Cancer. J. Nucl. Med..

[cit21] Corlett A., Sani M.-A., Van Zuylekom J., Ang C.-S., von Guggenberg E., Cullinane C., Blyth B., Hicks R. J., Roselt P. D., Thompson P. E. (2021). A New Turn in Peptide-Based Imaging Agents: Foldamers Afford Improved Theranostics Targeting Cholecystokinin-2 Receptor-Positive Cancer. J. Med. Chem..

[cit22] Corlett A., Pinson J.-A., Rahimi M. N., Zuylekom J. V., Cullinane C., Blyth B., Thompson P. E., Hutton C. A., Roselt P. D., Haskali M. B. (2023). Development of Highly Potent Clinical Candidates for Theranostic Applications against Cholecystokinin-2 Receptor Positive Cancers. J. Med. Chem..

[cit23] Saita Y., Yazawa H., Honma Y., Nishida A., Miyata K., Honda K. (1994). Characterization of YM022: its CCKB/gastrin receptor binding profile and antagonism to CCK-8-induced Ca2+ mobilization. Eur. J. Pharmacol..

[cit24] Seva C., Dickinson C. J., Yamada T. (1994). Growth-Promoting Effects of Glycine-Extended Progastrin. Science.

[cit25] Ashurst H. L., Varro A., Dimaline R. (2008). Regulation of mammalian gastrin/CCK receptor (CCK2R) expression in vitro and in vivo. Exp. Physiol..

[cit26] Haskali M. B., Roselt P. D., Binns D., Hetsron A., Poniger S., Hutton C. A., Hicks R. J. (2019). Automated preparation of clinical grade [(68)Ga]Ga-DOTA-CP04, a cholecystokinin-2 receptor agonist, using iPHASE MultiSyn synthesis platform. EJNMMI Radiopharm. Chem..

[cit27] Pawlak D., Rangger C., Peitl P. K., Garnuszek P., Maurin M., Ihli L., Kroselj M., Maina T., Maecke H., Erba P. (2016). From preclinical development to clinical application: Kit formulation for radiolabelling the minigastrin analogue CP04 with In-111 for a first-in-human clinical trial. Eur. J. Pharm. Sci..

[cit28] Maurin M., Garnuszek P., Baran P., Pawlak D., Mikołajczak R. (2015). The radiometal makes a difference. Synthesis and preliminary characterisation of DOTA-minigastrin analogue complexes with Ga, Lu and Y. Nucl. Med. Rev. Cent. East. Eur..

[cit29] Kolenc Peitl P., Rangger C., Garnuszek P., Mikolajczak R., Hubalewska-Dydejczyk A., Maina T., Erba P., Decristoforo C. (2019). Clinical translation of theranostic radiopharmaceuticals: Current regulatory status and recent examples. J. Labelled Compd. Radiopharm..

[cit30] Xu W., Chen G.-S., Shao Y., Li X.-L., Xu H.-C., Zhang H., Zhu G.-Q., Zhou Y.-C., He X.-P., Sun W.-H. (2013). Gastrin acting on the cholecystokinin2 receptor induces cyclooxygenase-2 expression through JAK2/STAT3/PI3K/Akt pathway in human gastric cancer cells. Cancer Lett..

[cit31] Gonzalez A., Santofimia-Castaño P., Salido G. M. (2011). Culture of pancreatic AR42J cell for use as a model for acinar cell function. Pancreapedia.

