# Correction: Evaluating the therapeutic efficacy of gastramide theranostics targeting cholecystokinin-2 receptors in a preclinical setting

**DOI:** 10.1039/d6ra90008a

**Published:** 2026-02-12

**Authors:** Marwa N. Rahimi, Jo-Anne Pinson, Joseph Hilton-Proctor, Jessica Van Zuylekom, Benjamin Blyth, Peter D. Roselt, Mohammad B. Haskali

**Affiliations:** a Department of Radiopharmaceutical Sciences, Cancer Imaging, The Peter MacCallum Cancer Centre Victoria 3000 Australia mo.haskali@petermac.org; b Sir Peter MacCallum Department of Oncology, The University of Melbourne Victoria 3010 Australia; c Models of Cancer Translational Research Centre, The Peter MacCallum Cancer Centre Victoria 3000 Australia

## Abstract

Correction for ‘Evaluating the therapeutic efficacy of gastramide theranostics targeting cholecystokinin-2 receptors in a preclinical setting’ by Marwa N. Rahimi *et al.*, *RSC Adv.*, 2026, **16**, 2123–2132, https://doi.org/10.1039/D5RA08789A.

The authors regret that chemical structures in Fig. 1 were incorrectly shown in the original article. The amended Fig. 1 is shown below with *N*-methylation of the C-terminal naphthylalanine residue for structures GA4 and GA13.

**Fig. 1:**
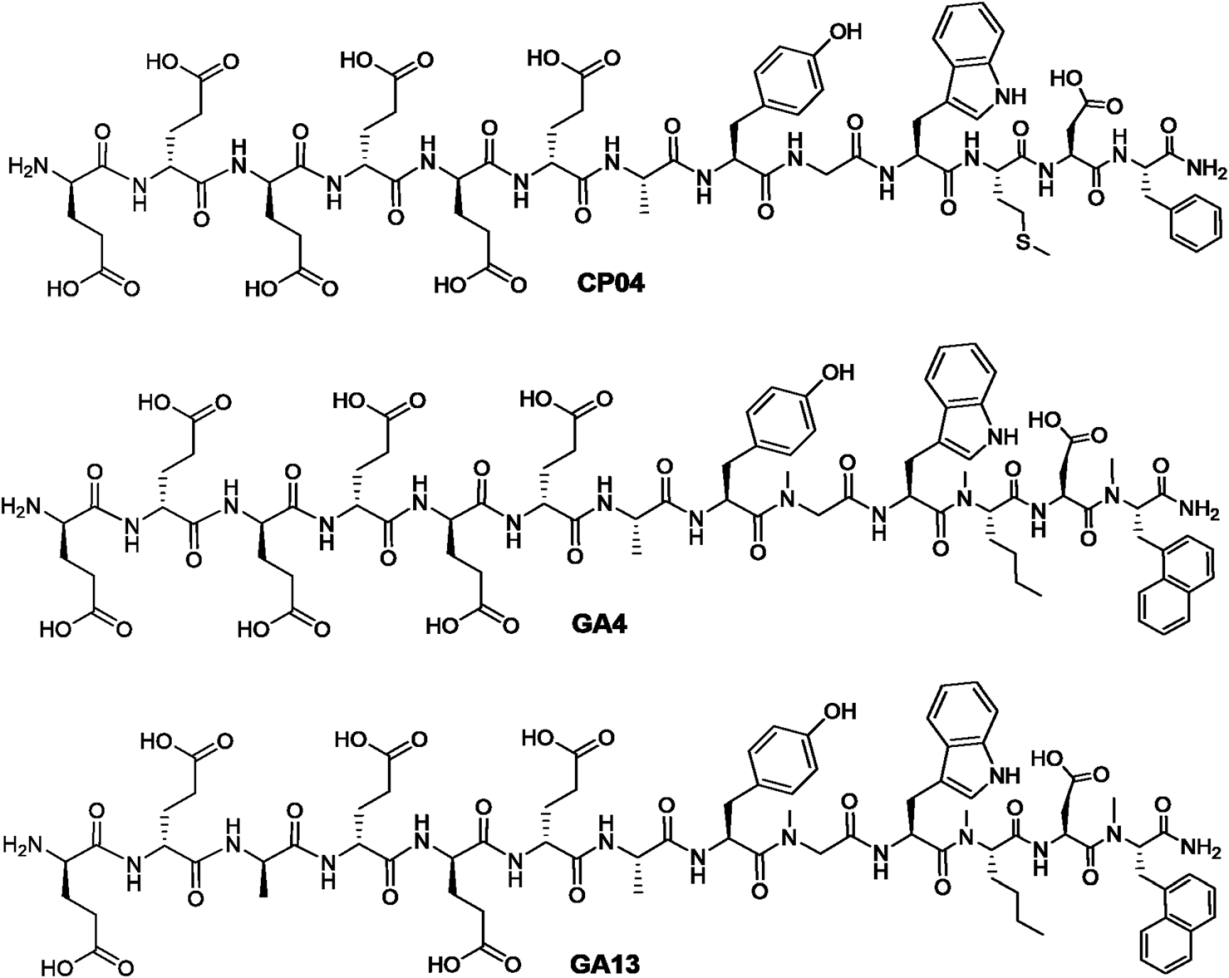
Chemical structures of CCK_2_R targeting peptides CP04, GA4, and GA13.

The Royal Society of Chemistry apologises for these errors and any consequent inconvenience to authors and readers.

