# Supplementary material for: Evaluating the therapeutic efficacy of gastramide theranostics targeting cholecystokinin-2 receptors in a preclinical setting
Source: RSC Adv. 2026 Jan 7;16(3):2123–32. doi: 10.1039/d5ra08789a (PMC12777951; doi:10.1039/d5ra08789a)
Supplement: RA-016-D5RA08789A-s001 [file RA-016-D5RA08789A-s001.pdf]

Supplementary Information (SI) for RSC Advances.
This journal is © The Royal Society of Chemistry 2025

Evaluating the Therapeutic Efficacy of Gastramide Theranostics Targeting Cholecystokinin-2 Receptors in a Preclinical Setting

Marwa N. Rahimi,[1,2] *Jo-Anne Pinson,*[1,2] *Joseph Hilton-Proctor,*[1,2] *Jessica Van Zuylekom,*[2,3] *Benjamin Blyth,* [2,3] *Peter D. Roselt,*[1,2] *Mohammad B. Haskali* [11,2]

[1] Department of Radiopharmaceutical Sciences, Cancer Imaging, The Peter MacCallum Cancer Centre Victoria 3000, Australia;

[2] Sir Peter MacCallum Department of Oncology, The University of Melbourne, Victoria 3010, Australia;

[3] Models of Cancer Translational Research Centre, The Peter MacCallum Cancer Centre, Victoria 3000, Australia;

Supplemental Materials

Corresponding author.

*E-mail: mo.haskali@petermac.org; ORCID: 0000-0003-3084-2084.

General Chemistry Methods:

Confirmation of peptide identity and purity as analyzed Analytical Radio-HPLC was performed using a Shimadzu HPLC system consisting of a SCL-10AVP system controller, SIL-0ADVP auto-injector, LC-10 ATVP solvent delivery unit, CV-10AL control valve, DGU-14A degasser, and SPD-10AVPV detector. This was coupled to a radiation detector consisting of an Ortec model 276 photomultiplier base with a 925-SCINTACE-mate preamplifier, amplifier, bias supply, SCA and a Bicron 1M11/2 photomultiplier tube. [177Lu]$LuCl_3$ was calibrated using the CRC-15PET dose calibrator (Capintec). This was calibrated daily using Cs-137 and Co-57 sources (Isotope Products Laboratories).

Radiochemical purity was determined using a Kinetex XB-C18 column (5 µm, 100 Å, 250 mm × 4.60 mm) with product eluted at 1 mL/min with a gradient of MeCN: 0.05% (v/v) TFA, commencing at 25% MeCN for 1 min, increased to 35% over 9 min, then immediately to 90%, maintained for 2 min, then returned to 25% MeCN. Specific activity (A_s) and molar activity (A_M) is determined at the end of synthesis (EOS).

Coupling of DOTA to peptides CP04, GA4 and GA13

DOTA (1.5 eq. relative to peptide) was pre-activated with *N*-hydroxysuccinimide (NHS) (2.25 eq.) using N-(3-dimethylaminopropyl)-N'-ethylcarbodiimide hydrochloride (EDCI) (2.25 eq.) and *N,N*-diisopropylethylamide (DIPEA) (3 eq.) in anhydrous dimethyl sulfoxide (DMSO) (500 µL). The reaction mixture was heated and gently sonicated at 50 °C for 30 min until starting material was dissolved. Peptides CP04, GA4 and GA13 (1 eq.) were dissolved in a minimum volume of anhydrous DMSO (~ 200–500 µL), then crude mixture was shaken for 30 min at room temperature. Reaction progress was monitored by LC-MS analysis, until completion was observed (usually

within 1 hour), observed by LC-MS analysis with the loss of the *m/z* peak of the starting peptide. Distilled water was added to form a 20:80 mixture of DMSO:water then crude peptide was purified using reverse phase HPLC.

Radiolabeling of peptides with ^{177}Lu

DOTA-peptides DOTA-CP04, DOTA-GA4, and DOTA-GA13 (30 µg, 14.6 nmol) were dissolved in a 0.5 M sodium acetate solution (100 µL, resulting in a peptide concentration of 0.3 µg/µL). This solution was then combined with 200 µL of 0.4 M ammonium acetate/0.24 M 2,5-dihydroxybenzoic acid (pH 4.5), along with 50 µL of ethanol and 50 µL of L-methionine (10 mg/mL solution in MilliQ water). Next, 500-1000 MBq of noncarrier-added [^{177}Lu]LuCl3 in 0.04 M HCl (50-100 µL) was added to the crude mixture, which was then heated to 80 °C for 30 minutes. This process yielded the ^{177}Lu-labeled peptides, [^{177}Lu]Lu-CP04, [^{177}Lu]Lu-DOTA-GA4, and [^{177}Lu]Lu-DOTA-GA13 (Figure S1), with radiochemical purity of ≥ 94% and quantitative yields (Table S1). All products showed over 90% purity by HPLC and TLC at 48 hours following the end of synthesis.

Table S1: Analytical data of [^{177}Lu]Lu-DOTA-CP04 , [^{177}Lu]Lu-DOTA-GA4 , [^{177}Lu]Lu-DOTA-GA13

Parameters	Acceptable specifications	[^{177}Lu]Lu-CP04	[^{177}Lu]Lu-DOTA-GA4	[^{177}Lu]Lu-DOTA-GA13
Appearance	Clear & colourless	pass	pass	pass
pH	4-8	5-6	5-6	5-6
Radiochemical purity (HPLC) [1]	≥ 90% labelled peptide	≥94%	99%	99%
Radiochemical purity (TLC)	≥ 98% labelled peptide	>98%	>98%	>98%
	≤ 2% free ^{177}Lu	<2%	<2%	<2%
Specific Activity	≥ 3.0 MBq/µg	16-35 MBq/µg	16-35 MBq/µg	16-35 MBq/µg

[1]HPLC conditions 1: Kinetex XB C18 column (5 µm, 100 Å, 250 × 4.60 mm) eluted at 1 mL/min with a gradient of MeCN: 0.05% (v/v) TFA, starting at 25% MeCN for 1 min, increased to 35% over 9 min and then instantly increased to 90% and maintained at that for 2 min then back down to 25% MeCN.

Radio-HPLC trace of [^{177}Lu]Lu-CP04

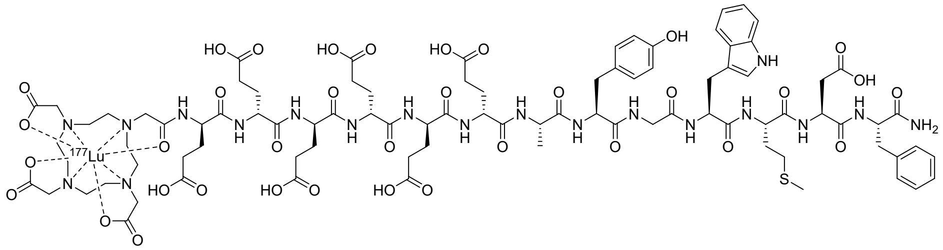

DOTA(^{177}Lu)-DGlu-DGlu-DGlu-DGlu-DGlu-DGlu-Ala-Tyr-Gly-Trp-Met-Asp-Phe-NH_2

Exact Mass: 2222.73
Molecular Weight: 2224.03

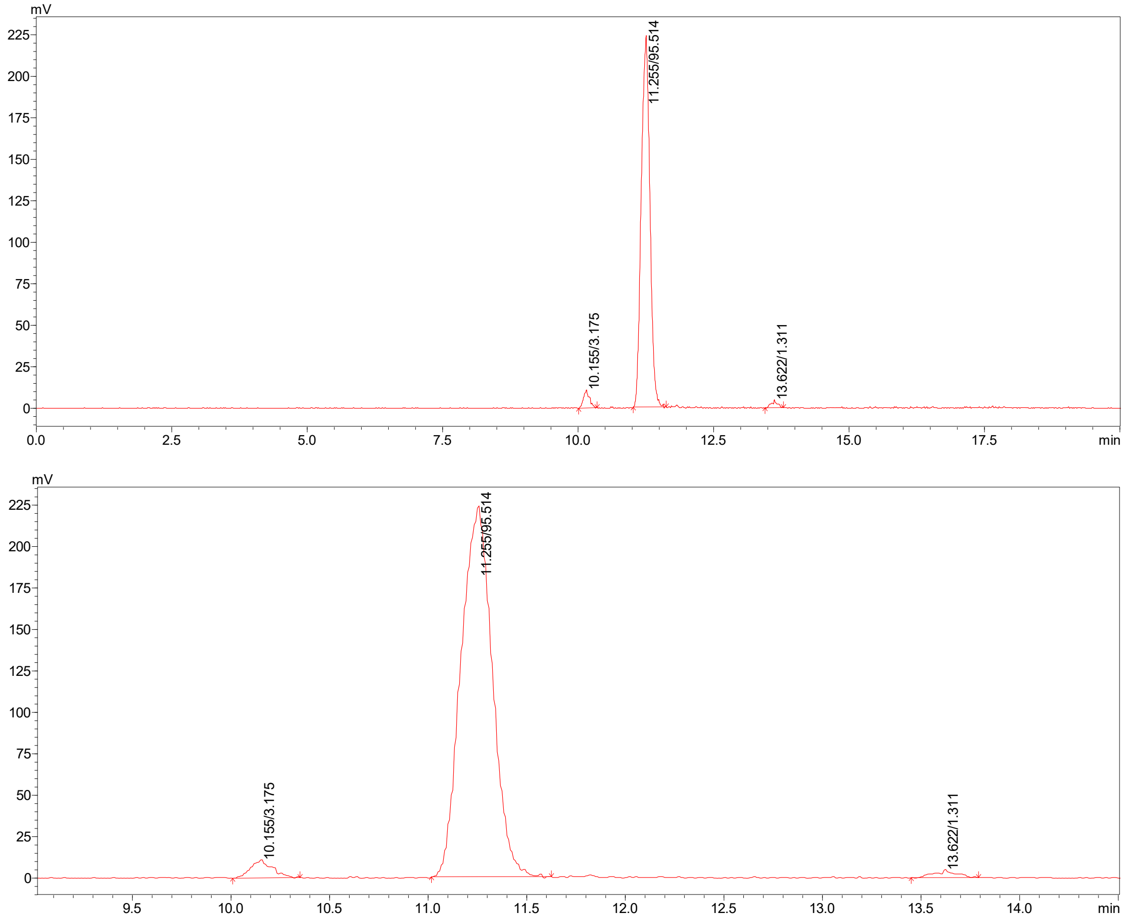

Figure S1: Radio-HPLC trace of [^{177}Lu]Lu-CP04.

Table S2: Peak retention time and AUC of [^{177}Lu]Lu-CP04 radio-HPLC.

Peak#	Ret. Time	Area	Area%
1	10.155	82125	3.175
2	11.255	2470725	95.514
3	13.622	33915	1.311
Total		2586764	100.000

Radio-HPLC trace of [^{177}Lu]Lu-DOTA-GA4

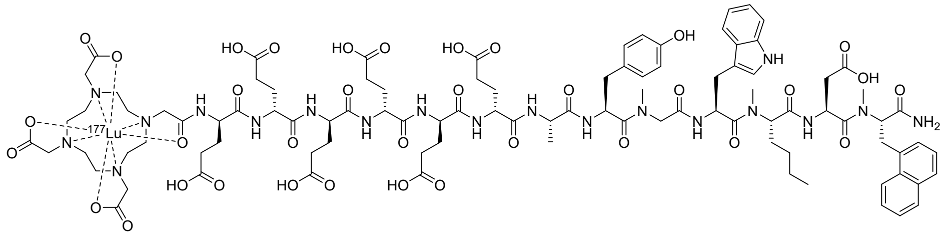

Exact Mass: 2295.83
Molecular Weight: 2297.13

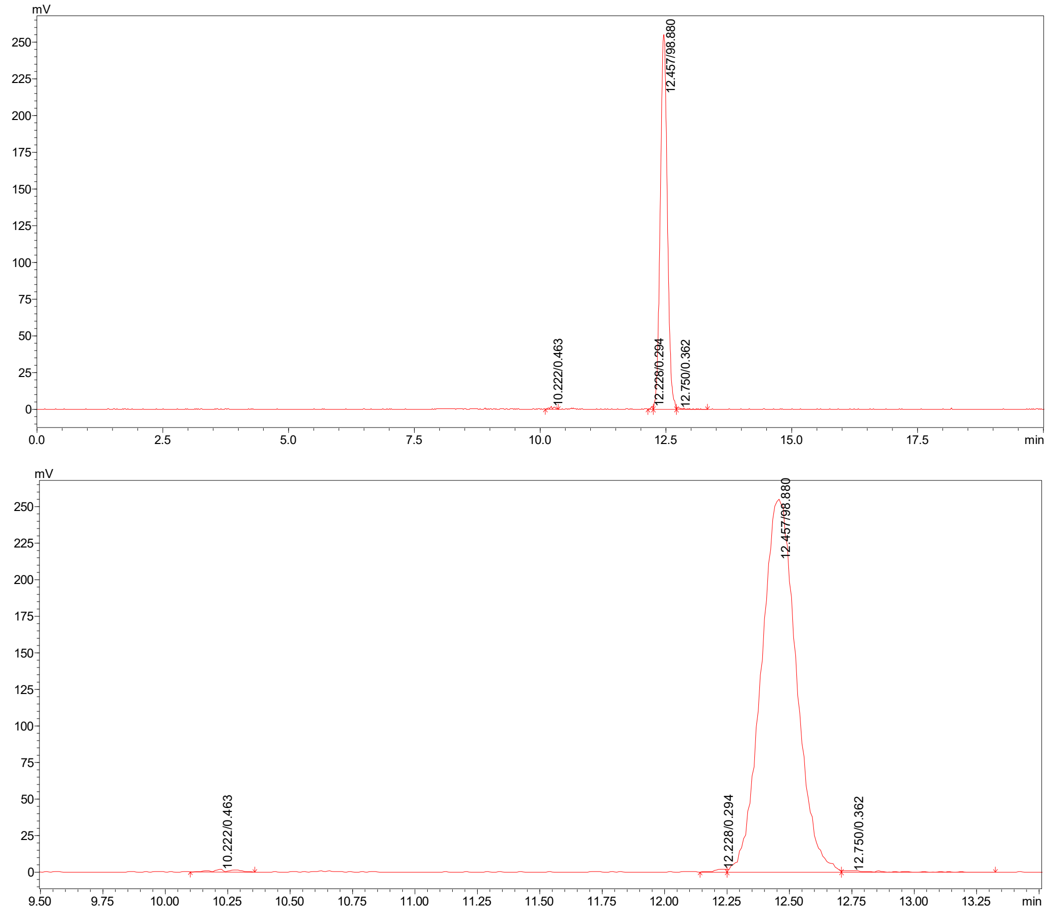

Figure S2: Radio-HPLC trace of [^{177}Lu]Lu-DOTA-GA4.

Table S3: Peak retention time and AUC of [^{177}Lu]Lu-DOTA-GA4 radio-HPLC.

Peak#	Ret. Time	Area	Area%
**1**	10.222	11659	0.463
**2**	12.228	7412	0.294
**3**	12.457	2488900	98.880
**4**	12.750	9112	0.362
**Total**		2517083	100.000

Radio-HPLC trace of [^{177}Lu]Lu-DOTA-GA13

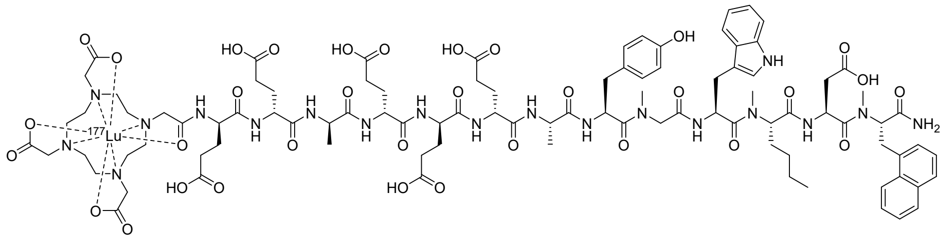

Exact Mass: 2237.82
Molecular Weight: 2239.10

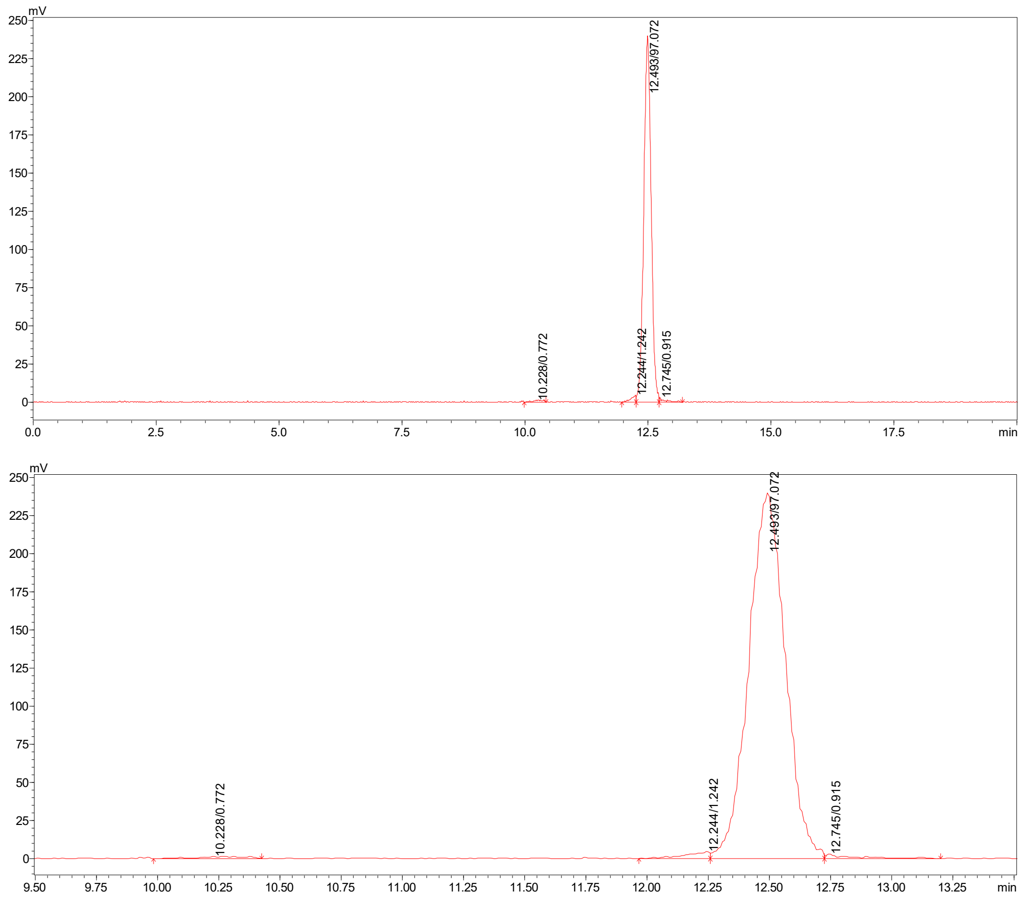

Figure S3: Radio-HPLC trace of [^{177}Lu]Lu-DOTA-GA13.

Table S4: Peak retention time and AUC of [^{177}Lu]Lu-DOTA-GA13 radio-HPLC.

Peak#	Ret. Time	Area	Area%
1	10.228	19791	0.772
2	12.244	31850	1.242
3	12.493	2489706	97.072
4	12.745	23468	0.915
Total		2564815	100.000

Radio-HPLC trace of [^{177}Lu]Lu-DTPA

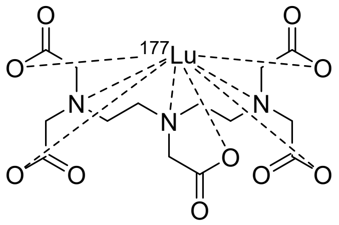

Exact Mass: 565.04
Molecular Weight: 565.25

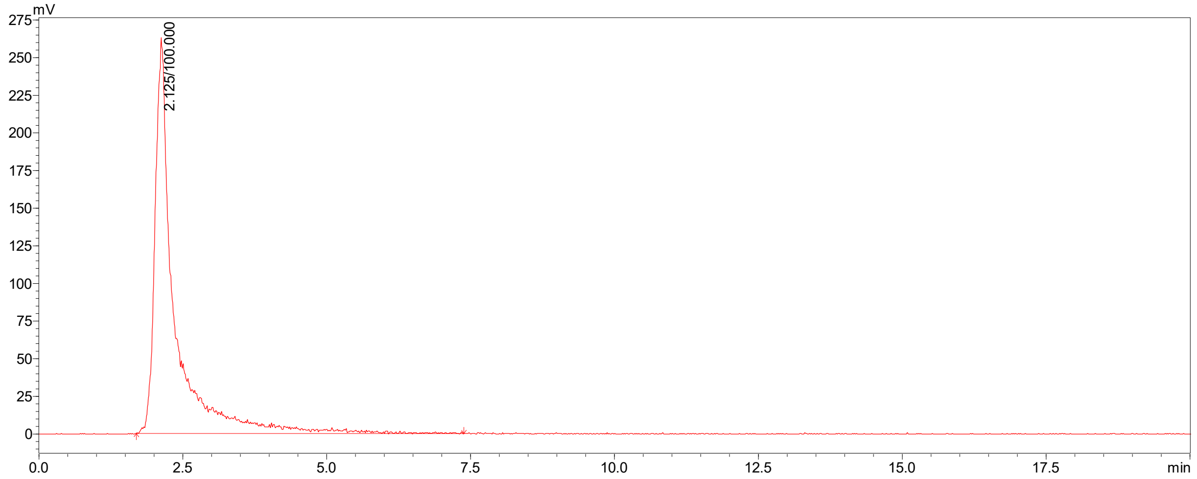

Figure S4: Radio-HPLC trace of [^{177}Lu]Lu-DTPA.

Mouse blood analysis: radio-HPLC traces of ligands at 15 minutes

Blood analysis: Metabolism of [^{177}Lu]Lu-CP04

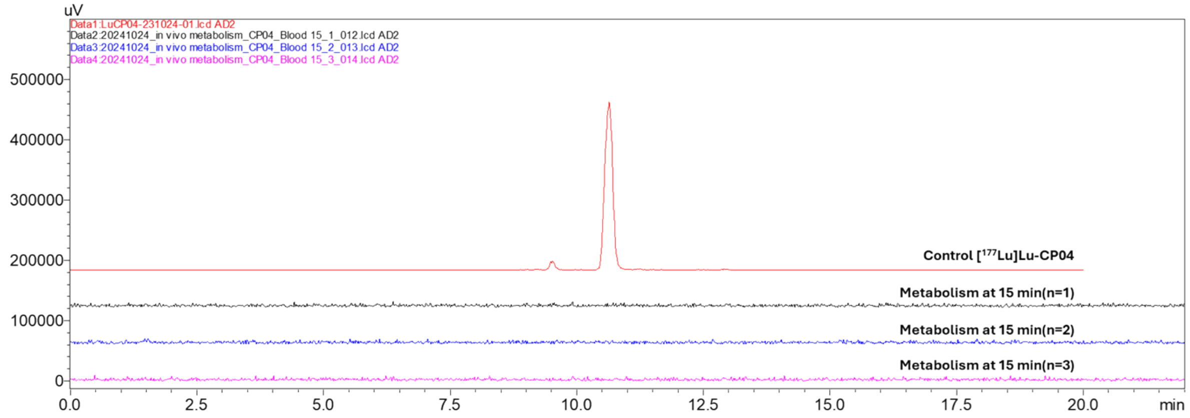

Figure S5: Overlayed radiochromatograms of control [^{177}Lu]Lu-CP04, and metabolized samples (n=3).

Blood analysis: Metabolism of [^{177}Lu]Lu-DOTA-GA4

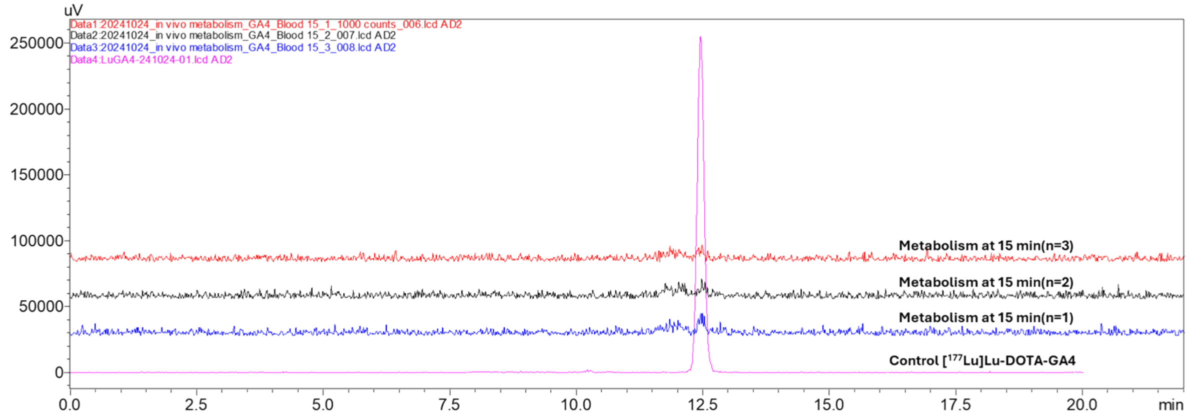

Figure S6: Overlayed radiochromatograms of control [^{177}Lu]Lu-DOTA-GA4, and metabolized samples (n=3).

Blood analysis: Metabolism of [^{177}Lu]Lu-DOTA-GA13

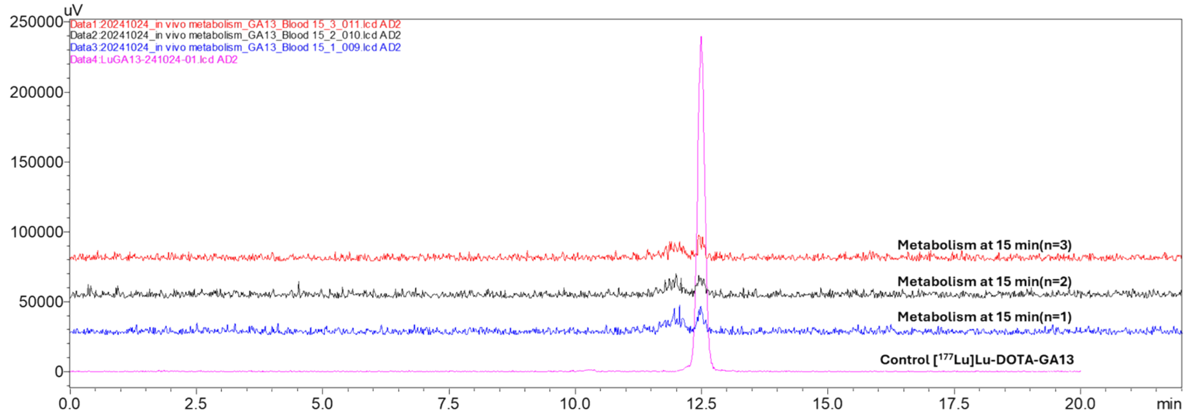

Figure S7: Overlayed radiochromatograms of control [^{177}Lu]Lu-DOTA-GA13, and metabolized samples (n=3).

Mouse urine analysis: radio-HPLC traces of ligands at 15 minutes

Urine analysis: Metabolism of [^{177}Lu]Lu-CP04

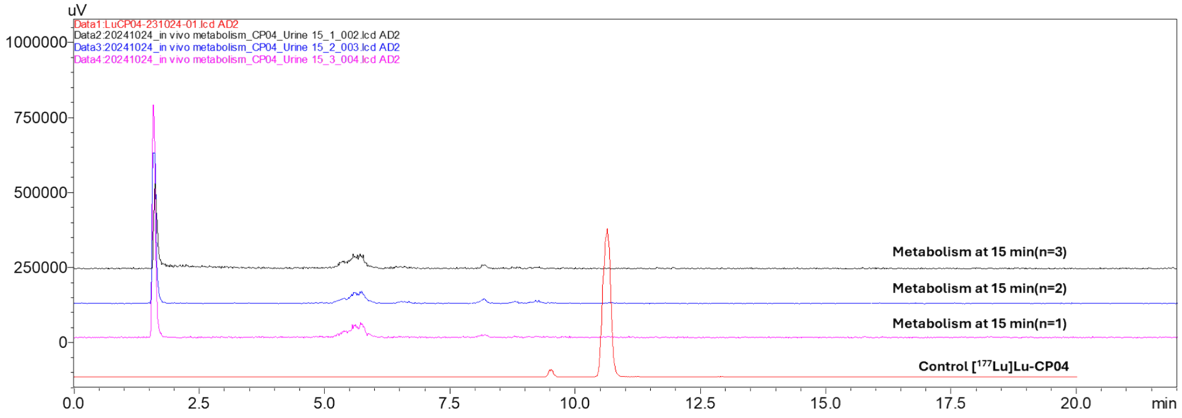

Figure S8: Overlayed radiochromatograms of control [^{177}Lu]Lu-CP04, and metabolized samples (n=3).

Urine analysis: Metabolism of [^{177}Lu]Lu-DOTA-GA4

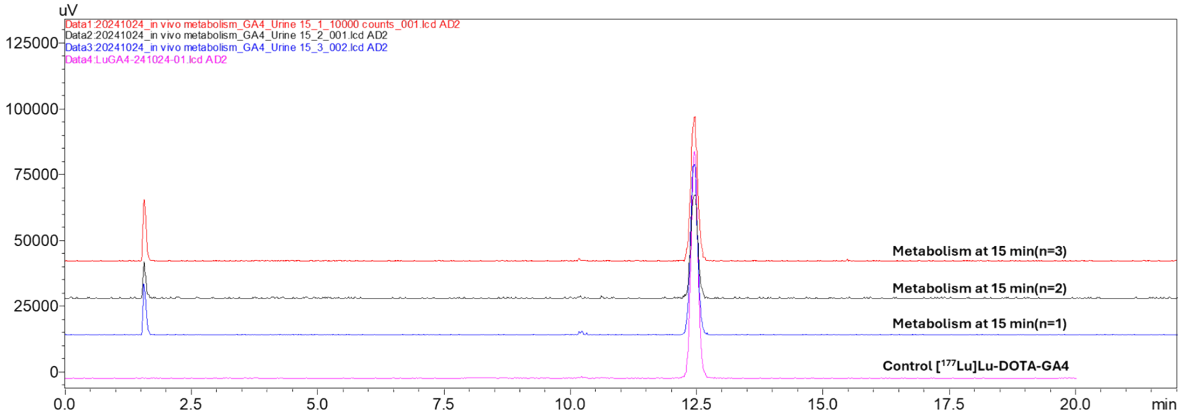

Figure S9: Overlayed radiochromatograms of control [^{177}Lu]Lu-DOTA-GA4, and metabolized samples (n=3).

Urine analysis: Metabolism of [^{177}Lu]Lu-DOTA-GA4

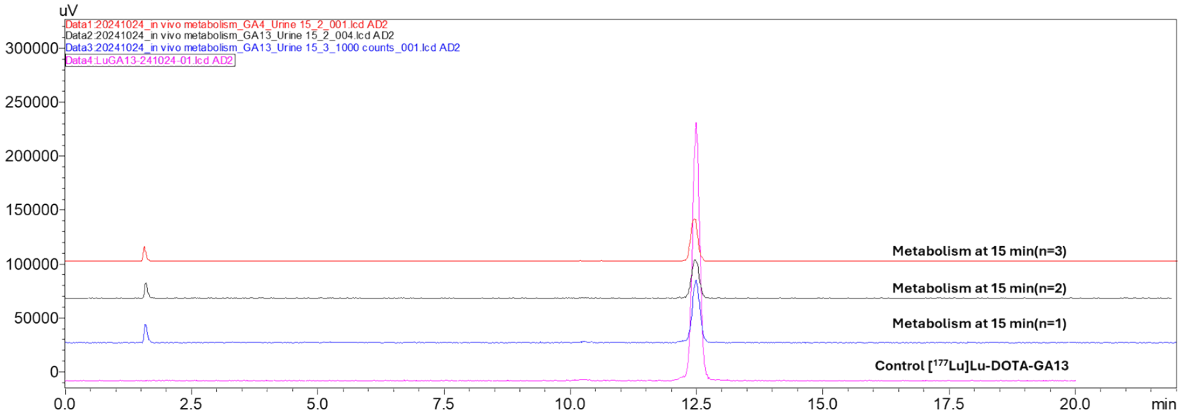

Figure S10: Overlayed radiochromatograms of control [^{177}Lu]Lu-DOTA-GA13, and metabolized samples (n=3).

Mouse weights measured for different treatments throughout the study

Table S5: Mouse weights during treatment with vehicle and 10 MBq [^{177}Lu]Lu-CP04, [^{177}Lu]Lu-DOTA-GA4 or [^{177}Lu]Lu-DOTA-GA13

Day	Vehicle						[^{177}Lu]Lu-CP04						[^{177}Lu]Lu-DOTA-GA4						[^{177}Lu]Lu-DOTA-GA13					
Mouse #	1	2	3	4	5	6	1	2	3	4	5	6	1	2	3	4	5	6	1	2	3	4	5	6
0	18.8	19.5	19.3	18.4	18.8	18	18.5	16.9	17	19.3	17.3	18	19.4	15.1	16.9	17.3	17.3	18.6	18.8	16.5	14.2	17	17.2	19.5
3	19.2	19.8	19.4	18.5	19.1	17.7	18.5	16.4	16.7	19	17.3	18	19.2	14.9	16.1	17.1	17.4	18.2	16.2	14.4	13.2	15.1	14.5	17
4																			18.4	16.3	14	17.1	16.6	19.4
6	20.5	19.9	20.2	19.2	20.7	18.8	19.5	17	17.3	20.2	18.1	18.3	19.1	14.8	16.3	17.5	17.4	18.9	18.5	16.5	14.4	17.3	16.7	20.2
8	20.7	20.8	21	19.6	20.8	19.4	20.5	17.6	18	20.4	18.8	18.8	19.5	15.3	16.7	17.8	18.2	19.5	18.6	17.1	15	18.2	17.1	21
9	21.4	21.4	21.1	19.6	21.1	19.6																		
10			21.1	19.6		19.7		17.6	18.1	20.3	18.4	18.7	19.4	15.5	16.5	18	18.7	19.6	18.7	17.3	14.9	17.9	17	20.4
13			21.2	20.4				17.8	19	21.2	18.7	19	19.6	15.9	17	18.3	18.8	19.6	18.6	17.3	15.3	18.5	17.7	21.1
15				21.3				18		21.4	19.3	19.3	19.8	16.3	17.8	19.2	19.2	20.1	19	17.7	17	19.3	18.6	21.6
17								18.6		21.9	19.3	19.8	20.1	16	17.3		19.3	20.7	19.2	17.6	15.8	18.8	17.7	21.3
20											19.1	20.5		16.4	17.4		19.8	20.6		17.7	16.2	19	17.4	21.7
22											19.8			17	17.8		20.4	21		17.9	16.3	18.9	17.9	22.8
23																	20.8	20.9		17.8				23.1
24											19.6			17	17.4						16.1	18.9	17.7	
27											20.1			18.5	18.4						16.6	19.5	18.4	
28											19.9				18.3									
29											20.5											20.3		
30											20.6													
31																						19.6		
34																						19.7		

Table S6: Mouse weights during treatment with vehicle and 20 MBq [^{177}Lu]Lu-CP04, [^{177}Lu]Lu-DOTA-GA4 or [^{177}Lu]Lu-DOTA-GA13

**Day**	**Vehicle**						**[^{177}Lu]Lu-CP04**						**[^{177}Lu]Lu-DOTA-GA4**						**[^{177}Lu]Lu-DOTA-GA13**					
**Mouse #**	**1**	**2**	**3**	**4**	**5**	**6**	**1**	**2**	**3**	**4**	**5**	**6**	**1**	**2**	**3**	**4**	**5**	**6**	**1**	**2**	**3**	**4**	**5**	**6**
0	18.8	19.5	19.3	18.4	18.8	18	16.9	20.4	19.3	17.6	17.4	19.9	19.7	18.8	19.0	18.0	16.0	18.4	18.4	18.2	18.8	17.7	17.9	18.0
3	19.2	19.8	19.4	18.5	19.1	17.7	17.2	20.3	18.8	17.6	17.8	19.5	17.9	18.9	19.0	18.0	15.9	18.1	18.7	18.1	18.2	17.2	18.5	18.4
6	20.5	19.9	20.2	19.2	20.7	18.8	17.3	20.6	19.1	18.0	17.9	19.6	18.1	19.2	18.9	18.2	16.1	18.3	18.8	18.4	18	17.3	18.2	18.6
8	20.7	20.8	21	19.6	20.8	19.4	17.9	21.1	19.3	18.4	18.2	20.1	19.0	19.7	19.1	18.8	16.5	18.8	19.6	18.9	18.6	17.7	19.2	19.5
9	21.4	21.4	21.1	19.6	21.1	19.6		21.3																
10			21.1	19.6		19.7	18.2		19.3	18.2	18.6	19.8	19.4	20.0	19.8	18.6	17.1	19	19.0	19.0	19.1	17.7	18.7	18.9
13			21.2	20.4			18.3		20.0	19.1	18.8	20.5	19.7	20.4	20.0	19.1	17.2	19.1	19.5	19.3	19.0	17.6	19.4	19.4
14							18.3																	
15				21.3			18.7		20.4	20.2	19.6	21.4	19.6	20.7	20.5	19.7	18	19.5	19.6	19.8	20.0	18.2	19.8	19.9
17									20.7		19.4	21.4	19.4	20.8	20.3	19.6	17.9	19.5	20.6	19.6	19.6	18.2	19.9	19.5
20									20.7			22.2	19.2	20.3	20	19.3	17.9	19.3	19.7	19.8	19.5	17.9	19.4	20.4
22									22.1			22.6	19.9	21.0	20.3	19.7	18.2	19.7	20.0	20.2	19.9	18.3	20.0	20.4
24													19.5	20.6	20.0	19.3	17.9	19.8	19.8	20	19.8	18.3	20.1	20.6
27													20.7	21.7	20.6	19.7	18.4	20.8	21.2	20.2	20	18.5	20.2	19.7
28																	19.1							
29													20.4	21.7	20.8	20.3			20.1	20.9	20.5	18.9	20.9	22.3
30																							20.5	
31													20.4	21.8	20.8	19.5			20.1	20.5	20.1	18.6		22.0
34													21.1		20.9	20.2			20.0	21.2	20.4	19.0		
35																					20.9			
36																					20.7			
38																					20.6			

Mean tumor volumes measured for each treatment throughout the study

Table S7: Mean tumor volumes (mm^3) at Day 0 and after treatment with 10 MBq of [^{177}Lu]Lu-CP04, [^{177}Lu]Lu-DOTA-GA4 or [^{177}Lu]Lu-DOTA-GA13. Values in red indicate mice close to endpoint. Values in blue indicate measurements excluded from median tumor measurements.

Day	Vehicle						[^{177}Lu]Lu-CP04						[^{177}Lu]Lu-DOTA-GA4						[^{177}Lu]Lu-DOTA-GA13					
Mouse #	1	2	3	4	5	6	1	2	3	4	5	6	1	2	3	4	5	6	1	2	3	4	5	6
0	205	164	119	67	127	75	190	214	127	70	95	51	108	139	60	225	69	137	303	155	127	70	61	98
3	380	391	190	144	454	172	484	181	205	148	114	38	270	260	61	583	112	266	438	243	208	78	89	145
6	780	694	332	302	672	377	724	334	433	181	179	58	450	207	90	602	112	252	524	388	345	89	134	139
8	1168	1014	434	333	956	412	1275	363	649	306	221	66	511	202	60	730	135	295	424	331	342	89	125	133
10			*899*	*409*		*1078*		*427*	*758*	*397*	*215*	*112*	447	217	88	654	170	346	456	382	246	71	112	182
13			*1438*	*817*				*627*	*1398*	*595*	*207*	*207*	617	269	66	943	197	334	706	348	168	129	90	205
15				*1204*				*914*		*899*	*313*	*335*	762	300	92	1284	316	427	725	340	268	116	107	245
17								*1361*		*1171*	*383*	*773*	*1096*	*406*	*147*		*362*	*802*	1099	513	273	147	163	374
20											*491*	*1478*		*413*	*205*		*662*	*613*		*548*	*343*	*232*	*264*	*644*
22											*508*			*581*	*318*		*906*	*936*		*877*	*611*	*320*	*435*	*968*
23																	*1329*	*1464*		*1232*				*1264*
24											*591*			*810*	*597*							*421*	*657*	
27											*1091*			*1612*	*1009*						*1350*	*687*	*1200*	
28											*930*				*1364*									
29											*1160*											*850*		
30											*1284*													
31																						*830*		
34																						*1267*		

Table S8: Mean tumor volumes (mm³) at Day 0 and after treatment with 20 MBq of [177Lu]Lu-CP04, [177Lu]Lu-DOTA-GA4 or [177Lu]Lu-DOTA-GA13. Values in red indicate mice close to the endpoint. Values in blue indicate measurements excluded from median tumor measurements.

Day	Vehicle						[177Lu]Lu-CP04						[177Lu]Lu-DOTA-GA4						[177Lu]Lu-DOTA-GA13					
Mouse #	1	2	3	4	5	6	1	2	3	4	5	6	1	2	3	4	5	6	1	2	3	4	5	6
0	205	164	119	67	127	75	127	304	143	117	71	43	60	86	105	128	162	233	113	151	134	58	192	79
3	380	391	190	144	454	172	247	498	300	196	87	47	72	133	136	171	253	314	177	270	218	145	314	142
6	780	694	332	302	672	377	253	813	121	285	161	50	55	131	90	248	199	249	211	237	280	111	426	139
8	1168	1014	434	333	956	412	487	1093	146	435	221	69	32	136	124	258	256	238	131	282	168	141	302	114
10			*899*	*409*		*1078*	*684*		*171*	*543*	*360*	*117*	64	118	141	223	220	249	154	207	190	89	309	99
13			*1438*	*817*			*1001*		*250*	*909*	*454*	*216*	29	100	70	140	178	196	183	285	188	120	347	101
15				*1203*			*1236*		*364*	*1357*	*689*	*215*	49	162	124	259	145	268	188	207	173	127	309	139
17									*527*		*1087*	*513*	69	137	110	258	215	373	159	231	198	146	319	132
20									*857*			*711*	49	189	136	218	253	408	273	237	162	161	392	266
22									*1430*			*1210*	225	338	157	387	401	514	325	334	230	187	400	289
24													238	283	127	318	564	495	226	317	238	235	333	208
27													506	609	299	473	1048	1257	696	489	211	375	879	525
29													*772*	*923*	*436*	*623*			465	571	360	512	1011	921
31													*853*	*1279*	*560*	*724*			*761*	*805*	*429*	*725*		*1329*
34													*1841*		*1239*	*1812*			*1417*	*1718*	*1145*	*1328*		
35																					*845*			
36																					*971*			
38																					*1241*			
